# Experience Versus Report: Where Are Changes Seen After Exposure-Based Cognitive-Behavioral Therapy? A Randomized Controlled Group Treatment of Childhood Social Anxiety Disorder

**DOI:** 10.1007/s10578-019-00954-w

**Published:** 2020-01-20

**Authors:** Julia Asbrand, Nina Heinrichs, Steffen Schmidtendorf, Kai Nitschke, Brunna Tuschen-Caffier

**Affiliations:** 1 Department of Psychology, University of Freiburg, Freiburg im Breisgau, Germany; 2 Department of Psychology, University of Bremen, Bremen, Germany; 3 Department of Psychology, University of Braunschweig, Braunschweig, Germany; 4 Department of Clinical Psychology and Psychotherapy, Institute of Psychology, University of Freiburg, Freiburg im Breisgau, Germany

**Keywords:** Group cognitive behavioral therapy, Social phobia, Treatment success, State measures

## Abstract

A considerable number of children and adolescents with social anxiety disorder (SAD) do not benefit from treatment as much as expected. However, treatment success should not be measured with social anxiety reports alone; the cognitive, behavioral, and physiological components of social stress should also be assessed. The authors examined an exposure-based SAD-specific group cognitive behavioral therapy (CBT) in a randomized controlled trial (*N* = 67, age 9–13 years, blind randomized allocation to treatment [CBT; *n* = 31] and waitlist control [WLC; *n* = 36] groups). Success was operationalized as a clinically significant reduction of symptoms measured with SAD-specific questionnaires, structured interviews, and changes in response to the Trier Social Stress Test (TSST). In the CBT group, there was a trend toward a significant increase in positive cognitions in the TSST after treatment (*d* = 0.37), whereas these positive cognitions decreased in the WLC group (*d* = 0.40). No significant results involving group appeared for negative cognitions, behavior and physiology. Children in the CBT group, but not parents, further reported less social anxiety in one questionnaire from pre- to post-treatment (*d* = 0.89). A structured interview confirmed a decrease in severity of SAD in the CBT group. While the gold standard of a blind interview showed efficacy of treatment, not all trait and state measures demonstrated similar success patterns.

*Trial registration* Eligibility criteria and some of the dependent variables (cognitions, physiology) for treatment success were registered with the German Research Foundation (TU 78/5-2, HE 3342/4-2) prior to recruitment. Clinical assessment of diagnosis and behavioral data were not a priori planned as outcome measures for this trial and therefore analyzed in a post-hoc approach.

**Electronic supplementary material:**

The online version of this article (10.1007/s10578-019-00954-w) contains supplementary material, which is available to authorized users.

## Introduction

Social anxiety disorder (SAD) is one of the most common mental disorders associated with great impairment in the well-being and everyday life of affected children and youth [1]. With prevalence rates as high as 9% in youth [2], effective and efficient treatment is essential. Cognitive behavioral therapy (CBT) programs have generally proven effective for anxiety disorders in children, adolescents, and adults [3, 4]. For example, typical programs such as the Coping Cat program consists of identification of anxious feelings, cognitive restructuring, positive self-talk and exposure tasks, as well as rewards for efforts to cope with anxiety. However, in comparison to other anxiety disorders, a primary or comorbid diagnosis of SAD usually leads to less remission of symptoms and lower response rates in generic treatment programs [4-7]. Treatments based on theoretical models of SAD [4] and tailored to SAD-specific deficits may be needed. Furthermore, in contrast to the more common approach of measuring treatment success by reductions in SAD symptoms and decreases in the severity of a clinical diagnosis, additional relevant measures (e.g., experiences in social situations), should be taken into account.

## SAD-Specific Treatment

As the core symptom of SAD is fear of social interactions often combined with difficulty performing adequately in social situations, a treatment specific to SAD may benefit from including peers to enhance possibilities for social interaction with peers in treatment (e.g., to provide continuous exposure). In addition to changing the treatment’s content (i.e., focus on cognitions), changing the treatment’s structure from individual to group may therefore likely be a second important adaptation to target SAD specifically. Group CBT programs have gained influence as a generic treatment approach for child and youth anxiety [8, 9]. In children and youth suffering from different anxiety disorders, group CBT has been shown to achieve benefits similar to those of individual CBT, and these have remained stable at 1-year follow-up [10]. A group approach allows almost constant exposure to other individuals and direct feedback from interaction partners. Interestingly, only few studies have focused on group treatment targeting SAD in adolescence [11-17]. Some pilot studies of small samples showed significant reduction in social anxiety symptoms after group treatment in adolescence [11, 12, 17]. A direct comparison of individual versus group therapy for SAD did, however, not result in a clear preference for either [13, 14, 18].

To date, even though the earliest onset of SAD has been reported at age 7 [19] to 9.2 years [2], almost all group CBT programs have been developed for adolescents starting at 12 years of age. Importantly, Halldorsson and Creswell [20] point out that preadolescents differ developmentally from adolescents. Only a few group treatments have focused on SAD in children [21, 22]. These studies showed substantial and stable therapeutic effects, but a large number of patients did not respond to the treatment. Thus, therapeutic effects may be enhanced if treatment programs include more exposure, which has been confirmed as the method of choice for adult patients with anxiety disorders [23-26]. The above mentioned treatments only used a low level form of exposure during social skills training, as homework [22] or as a short element in combination with cognitive restructuring [27]. Current studies suggest that exposure therapy is a key element in changing cognitions as negative expectations are challenged, attention biases corrected and positive cognitions applied [23].

## SAD-Specific State Assessment of Treatment Success

CBT is based on the assumption that affective, cognitive, behavioral, and physiological responses are highly interrelated and are, thus, the basis for both psychopathological symptoms and treatment (e.g. [28].). Therefore, assessment of all responses would appear crucial. Rather than targeting these responses individually, as has been done in previous studies, a social stress task including public speaking (Trier Social Stress Test for Children; TSST-C [29]) could be used to evaluate these responses in SAD, as it induces disorder-similar stress. Concerning affective arousal, children with SAD report more social anxiety during social stress (e.g. [30].). While this heightened state anxiety is already apparent at baseline (i.e., anticipation anxiety), it increases during stress but decreases during recovery, showing a modulation back to baseline levels. Regarding cognitive responses, children with SAD report more negative post-event processing, that is, negative thoughts about their own failings after having experienced a social situation (e.g.[31], and negative anticipatory cognitions concerning an upcoming social situation (e.g.[32]. Concerning behavioral symptoms, children with SAD usually report a more negative perception of their social skills; that is, they perceive their own actions as more nervous and believe they make a negative impression (e.g. [33].). Finally, results on physiological arousal show a tonic hyperarousal during social situations that can be seen in both heart rate levels and electrodermal activity (e.g. [30].). Only few studies have addressed these variables as possible treatment outcome variables: Adult studies have shown that cognitions in SAD change as a result of CBT [34], but results are inconclusive about changes in heart rate [35]. Regarding behavior, an increase in parent-perceived social skills in children after CBT was shown [22]. Examining all aspects of the CBT model together might be useful for measuring treatment success.

## The Current Study

Taking these findings into account and in line with recent work on exposure (e.g. [23].), we previously tested a SAD-specific exposure-based group treatment in a randomized controlled trial with 74 children (aged 8 to 12 year) with SAD [36]. Compared to parents of children in a waitlist control (WLC) group, parents of children in a CBT group reported a greater decrease in symptoms (CBT: *d* = 1.02, WLC: *d* = 0.06), but children did not differ on two measures of social anxiety. Still, an estimate of total treatment effects showed a steady decrease in social anxiety symptoms with medium to large effect sizes reported by both parents and children [36]. It is well known that parent–child agreement on (specific) anxiety disorders such as SAD [37] or anxiety symptoms [38] is low to moderate only. Although a meta-analysis also reported moderate to large agreement [39], the agreement on social anxiety in single studies is modest at best (e.g. [40-42].). This implies that treatment success may need to take both child and parent perspectives into account [41]. Additionally, to allow for a more differentiated picture after treatment, in this current study we have included both a structured interview and a social stress task to evaluate if state social anxiety (cognitions, behavior, physiology) changes even if this is not reflected in social anxiety reports.

For these reasons, we aimed to examine the effects of exposure-based CBT on children with SAD with both reports of social anxiety and an assessment of social anxiety during a laboratory task. The study was designed as a randomized controlled trial, in which half of the participants were allocated to an experimental group (CBT) receiving immediate treatment and the other half to a waitlist control (WLC) group receiving therapy about 16 weeks later. We tested laboratory and diagnostic data: We expected that compared to the WLC group and the first TSST-C before treatment, children in the CBT group would (a) report more positive and fewer negative cognitions (measured by the Social Interaction Self-Statement Test-Public Speaking, SISST-PS; [43]), (b) perceive their performance as less nervous (measured by the Performance Questionnaire for Children, PQ-C; [44]), and (c) show a change in heart rate. We did not expect differences concerning the affective part of social stress as the TSST-C is a very strong stressor, even inducing high social anxiety in nonclinical samples [45]. Further, (d) two different measures for children were used to examine a decrease in self-reported social anxiety symptoms in the CBT group after receiving treatment (questionnaires). This effect was expected to be confirmed by (e) parent report (questionnaire) and (f) a decrease in the severity index of a clinical diagnosis (interview).

All questionnaire measures were assessed at admission, pre-treatment/waiting, and post-treatment/waiting. Interview and laboratory measures were assessed at admission and post-treatment/waiting. A secondary analysis of stability of treatment effects is reported in the online supplements (S1). In addition, we exploratively assessed self-focused attention and emotion regulation. The pre-post results for these measures may be found in the online Supplements (S2).

## Method

### Trial Design

**Fig. 1:**
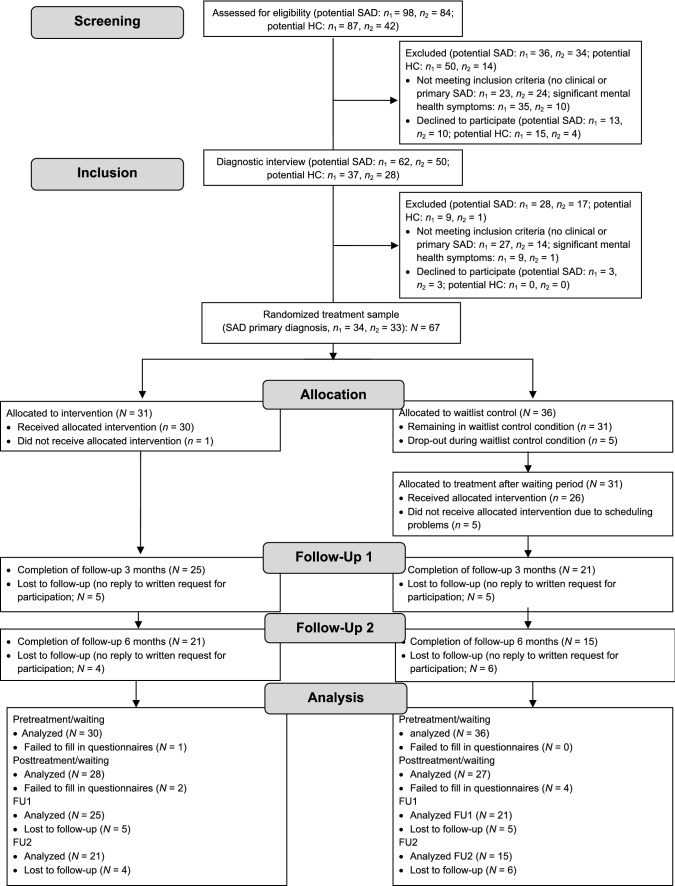
Flowchart of study participants for diagnostic data. *SAD *social anxiety disorder, *HC* healthy controls, *n*_*1*_ research center 1, *n*_*2*_ research center 2, *FU* follow-up. Analyzed data refer to questionnaires. Final sample sizes for all other analyses may vary due to single missing data points. Further detail is provided in the Method section. Results from the follow-up analyses are reported in the online supplements. Note: a healthy control group was recruited to address issues not covered in this manuscript (see Trial design) and is listed here for the sake of completeness

For this randomized controlled trial we used block randomization, in which about half of the participants were allocated by drawing from a hat to an experimental condition receiving immediate treatment and half to a WLC condition receiving treatment about 16 weeks later (for an overview see Fig. 1). Randomization for each of two research centers was conducted in a concealed fashion by the other center, based on subject codes, as soon as there were enough participants for one experimental and one WLC allocation. Eligibility criteria were specified and registered with the German Research Foundation (TU 78/5-2, HE 3342/4-2) prior to recruitment and were not changed during the study. Due to narrative considerations, some of the primary outcome variables are reported elsewhere.^1^ The current study reports state anxiety, negative cognitions, and physiological arousal as primary outcome variables. The sample size for laboratory data was determined based on a power analysis (*f* = 0.25, 1 − β = 0.80) and set at *N* = 54. For diagnostic data, a smaller effect size (*f* = 0.15, 1-β = 0.80) resulted in a requirement of *N* = 62. As the study was part of a larger research project requiring a larger sample, all children involved in the larger study were included to increase power.

### Participants

Families with anxious children (age 9 to 13 years) were recruited through advertisements in schools and medical facilities and through newspaper articles in two midsized German cities from January 2012 to November 2013 until the targeted sample size (plus 25% to face possible loss of data) had been reached (for an overview see Fig. 1). Follow-ups ended in June 2014. No harms were reported. The treatment trial was part of a larger project, which is presented elsewhere (see footnote 1). In compensation for participation in the laboratory study, parents received €35, and children €25 in vouchers. An independent ethics committee (ethics committee of the German Society for Psychology) granted ethics approval for this study. All participating children and their caregivers consented in oral and written form.

Demographics and psychometric measures are reported in Table 1. The groups did not differ in age, type of school, or any of the disorder-specific measures. Scores on both the Social Phobia and Anxiety Inventory for Children (SPAI-C) and the Social Anxiety Scale for Children–Revised (SASC-R) exceeded suggested cut-offs for clinically relevant SAD.

**Table 1:** Participant characteristics

Variable	Experimental group (CBT) *n* = 31			Control group (WLC) *n* = 36			Statistics
	*M*	*SD*	%	*M*	*SD*	%	
Age (in years)	11.5	1.35		11.2	1.33		*t*(65) = 0.84, n.s
Female			51.6			67.6	χ^2^(2) = 1.88, n.s
Comorbid diagnoses			41.9			45.9	χ^2^(2) = 1.66, n.s.
Primary school			29.0			32.4	χ^2^(4) = 0.73, n.s.
SASC-R (child report)^a^	49.8	13.98		49.5	12.84		*t*(64) = 0.81, n.s.
SASC-R (parent report)^b^	60.5	12.55		60.7	9.39		*t*(64) = 0.09, n.s.
SPAI-C	21.9	10.20		23.7	7.74		*t*(65) = 0.83, n.s.
Kinder-DIPS severity^c^							χ^2^(3) = .3.77, n.s.
Impaired (4)			12.9			29.7	
Moderately impaired (5)			64.5			54.1	
Clearly impaired (6)			19.4			16.2	
Severely impaired (7)			3.2			0.0	

*CBT* cognitive behavioral therapy, *WLC* waitlist control, *SASC-R* Social Anxiety Scale for Children-Revised (cut-offs: 50 for boys, 54 for girls; La Greca & Stone, 1993), *SPAI-C* Social Phobia and Anxiety Inventory for Children (cut-off: 18 for boys and girls; Beidel, Turner, & Morris, 1995), *Kinder-DIPS* Diagnostic Interview for Mental Disorders in Childhood and Adolescencem, *n.s.* not significant

^a^Missing data *n*_CBT_ = 1, *n*_WLC_ = 0

^b^Missing data *n*_CBT_ = 0, *n*_WLC_ = 1

^c^Severity index: 0 (*no impairment*) to 8 (*very severe impairment*)

### Procedure

#### Diagnostic Procedure

The study took place at two German universities.^2^ After a short telephone screening for anxiety symptoms, eligible children and their parents attended a diagnostic session (see flowchart in Fig. 1). Diagnoses of SAD and comorbid disorders (*Diagnostic and Statistical Manual of Mental Disorders*, 4th ed., text rev.; [46]) were reached by combining individual structured clinical interviews with both the child and a parent separately using the Diagnostic Interview for Mental Disorders in Children and Adolescents (Kinder-DIPS) supervised by an experienced clinical psychologist [47]. Trained interviewers conducted all diagnostic sessions. Additionally, children and parents reported sociodemographic data, anxiety symptoms, and general psychopathology in online questionnaires. On the basis of the diagnostic assessment, 67 children fulfilled the inclusion criterion of a primary diagnosis of SAD. Exclusion criteria included health problems (e.g., asthma, cardiac arrhythmia) and medication (e.g., methylphenidate) that could have interfered with psychophysiological assessment.

Participants were randomized to the CBT or WLC group. The assessments took place in parallel for the CBT and WLC groups before (pre-treatment/waiting) and after (post-treatment/waiting) a 12-session therapy program for the CBT group or waiting period for the WLC group. Children in the WLC group received treatment after the post-treatment assessment. To ensure similar periods between assessments, questionnaires assessing psychopathology were administered not only after the interview (admission) but also directly before (pre-treatment/waiting) as well as directly after (post-treatment/waiting) completion of the treatment or waitlist period.

After the child had either attended the CBT or waited for treatment in the WLC group, all children and parents were again asked to report on the child’s social anxiety symptoms using the same diagnostic questionnaires. Additionally, all parents were interviewed by a trained interviewer who was blind to the child’s treatment status using the disorder-specific SAD section of the Kinder-DIPS. Children in the WLC group received treatment after the second interview. Treatment effects were assessed with the same set of questionnaires after 3 months (Follow-Up 1) and after 6 months (Follow-Up 2).

#### Laboratory Procedure

**Fig. 2:**
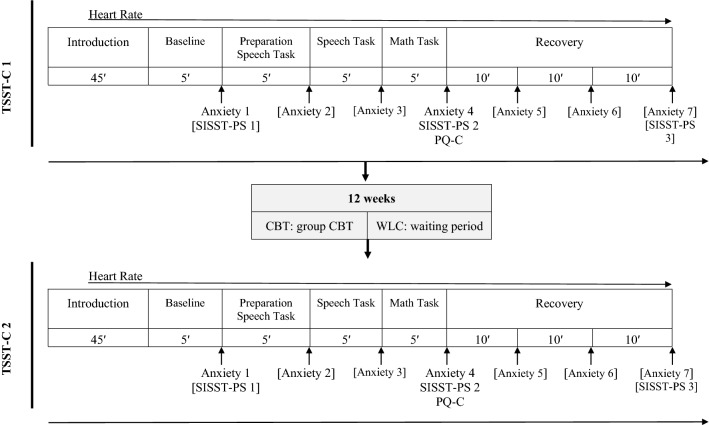
Overall procedure including the Trier Social Stress Test for Children (TSST-C) before (TSST-C 1) and after (TSST-C 2) treatment or waiting. Analyzed measures are indicated by omission of brackets. *CBT* group receiving, *WLC* waitlist control, *SISST-PS* Social Interaction Self-Statement Test-Public Speaking, *PQ-C* Performance Questionnaire, *Anxiety* children’s self-rating of anxiety

Following the diagnostic interviews, children participated in the first TSST-C [29], consisting of a speech and a math task (see Fig. 2). All assessments were conducted mid-afternoon (between 3 and 6 pm). Children were seated throughout the assessment. In the speech task, children were asked to continue narrating a story in front of two observers after listening to the beginning of the story. In the following mental arithmetic task, children were asked to serially subtract the number 7 from 758 (9- to 11-year-olds) or the number 13 from 1023 (12- to 13-year-olds) as fast and as accurately as possible. Both observers were instructed and trained to give neutral verbal and nonverbal feedback. Heart rate was assessed throughout the session; positive and negative cognitions (SISST-PS; [43]) were assessed (a) before the social stress task, (b) directly after the task, and (c) during recovery (see Fig. 2). Self-rated social performance was assessed after the social stress task. To allow a broad assessment of the height of social stress itself, only this latter time of measurement was included in the analyses, i.e. self-assessments about the speech and math task as well as heart rate during the speech and math task (5 min each) were used in the analyses. Additionally, children rated their anxiety using developmentally appropriate 11-point Likert-type scales taken from the scales of the Iconic Self-Assessment of Anxiety in Children [48]. All anxiety ratings referred to the maximum anxiety in the last period. For anxiety analyses, we used anxiety at baseline (anxiety_base_; Anxiety 1 in Fig. 2) and anxiety directly after stress (anxiety_stress_; Anxiety 4 in Fig. 2). After participating in a 12-week CBT program (CBT group) or waiting without treatment (WLC group), all children performed a parallel version of the first testing session (TSST-C 2 in Fig. 2; [29]), but the speech task was changed to a different story that was evaluated to be similarly interesting and difficult in a pre-evaluation. The math task was changed to a different start number (+ 10). The TSST-C reliably induces social anxiety in all children, even more so in children with SAD compared to healthy control children, *p* < 0.001.^3^

### Treatment

We applied an exposure-based CBT according to a manual [49] based on Clark and Wells’s [50] cognitive model of social phobia. It contains exposure as the main treatment mechanism (cf [23]), which has been described in more detail elsewhere and has shown promising improvement of symptoms [36]. In comparison to earlier research [21, 22] some (indirect) exposure characteristics are similar: First, the group itself provides an entry-level stage of exposure. Second, social skills training is a preliminary step to exposure. In our application, this social skills training is also used as a first exposure to challenging situations involving social. Finally, several sessions are reserved to conduct exposure in vivo outside of the therapy room (e.g., at the train station, in the city, etc.). Intervention components consist of psychoeducation, cognitive restructuring, social skills training, exposure, and relapse prevention. Children first learn about the nature of anxiety in general and its different components (cognitions, physiological arousal, and behavior). Understanding the relation between cognitions and anxiety is key for the cognitive restructuring, which uses SAD-specific cognitions. During social skills training, children are exposed to socially challenging situations individually selected on the basis of their fear hierarchy. Exposure sessions also relate to individual social fears (e.g., approaching a man with a beard to ask for directions). Relapse prevention is geared toward specific upcoming social challenges. We conducted 12 sessions (100 min each including a 10-min break) in groups of five to seven children.

### Psychometric Measures

#### Diagnostic Interview for Mental Disorders in Children and Adolescents (Kinder-DIPS)

The Kinder-DIPS [47] is a validated interview for the most frequent mental disorders in children and youth and is an extended and modified version of the Anxiety Disorders Interview Schedule for children (ADIS-C) [51]. The diagnosis is based on both child and parent reports. The authors report adequate interrater reliability (87% for anxiety disorders), good test–retest reliability, and successful validation with disorder-specific questionnaires. Parents and children agree only moderately on psychopathological symptoms on the Kinder-DIPS [52], similar to findings on the ADIS-C [53]. Children met diagnostic criteria if the severity rating was 4 or higher on a scale of 0 (*no impairment*) to 8 (*very severe impairment*).

#### Social Anxiety Scale for Children-Revised (SASC-R)

The SASC-R [54] measures social anxiety as reported by children and their parents (18 items, e.g., “I get nervous when I talk to new kids”). It consists of two sub scales Fear of Negative Evaluation (FNE) and Social Avoidance and Distress (SAD). Children and parents respond to each item using a 5-point Likert-type scale ranging from 1 (*not at all*) to 5 (*all the time*). The authors of the German version [55] reported acceptable test–retest reliability (0.67) and internal consistency (0.76). Moderate correlations have been confirmed with general measures of anxiety, self-perception of social confidence, teacher ratings of anxiety withdrawal, and peer nominations of popularity [56]. The internal consistency of the SASC-R in the current sample was excellent (child report: α = 0.95, parent report: α = 0.97).

#### Social Phobia and Anxiety Inventory for Children (SPAI-C)

The SPAI-C [57] assesses behavioral characteristics specific to SAD (26 items; e.g., “I am anxious when I meet new boys or girls”). Nine of the 26 items include sub-items assessing differences in level of anxiety by audience type (boys and girls I know, boys and girls I don’t know, adults). Children respond to each item using a 3-point Likert-type scale ranging from *never or hardly ever* to *almost always or always*. Validity and reliability were confirmed in the original sample [57] and a German sample [58]. Internal consistency and test–retest reliability after 4 weeks in the German sample were excellent (Cronbach’s α = 0.92; *r*_tt_ = 0.84). The internal consistency of the SPAI-C in the current sample was excellent (α = 0.98).

#### Social Interaction Self-Statement Test-Public Speaking (SISST-PS)

An adapted version [59] of the original SISST-PS [43] assesses eight positive (e.g., “I feel pretty good about my performance”) and eight negative (e.g., “What I say will probably sound stupid”) self-statements. Items are assessed on a 4-point Likert-type scale ranging from 0 (*never*) to 4 (*very often*). Scores on the subscales can range from 0 to 24. In the current study, Cronbach’s alpha was 0.83 for the positive cognitions subscale and 0.92 for the negative cognitions subscale.

#### Performance Questionnaire for Children (PQ-C)

The PQ-C [60] comprehensively assesses three aspects of social performance: microbehaviors (three items, e.g., “How loud and clear was your voice?”), nervousness (two items, e.g., “How nervous did you look?”), and global impression (three items, e.g., “How friendly did you look?”) by self-report. Items are scored on a 4-point scale ranging from *not very (much)* to *very (much).* As suggested by Cartwright-Hatton and colleagues (2005) [60], some minor changes were made to the original to adjust the questionnaire to the setting: “How much did you look at the camera?” was replaced with “How much did you look at the person you were talking to?” In addition, one further item relating to nervousness was added—“How much did you blush?” In the study, the German translation was used [61]. Internal consistency of the PQ-C was excellent (α = 0.94).

#### Heart rate

We assessed electrocardiograms at 400 Hz using the Varioport system (Becker Meditec, Karlsruhe, Germany). Data inspection and artefact rejection were performed offline using ANSLAB [62]. For the electrocardiograms, the cardiac interbeat interval, calculated as the interval in milliseconds between successive R waves, was extracted. For presentation of results, IBI was converted to HR, while IBI values were used in all statistical analyses [63]. As artefactual R-spikes in the ECG are likely to bias estimates of HR variability parameters, they were standardized by manual interpolation and deletion [64].

### Statistical Analysis

#### Laboratory Data

For the main analyses of^4^ all laboratory data, IBM SPSS Statistics (version 24) was used. For treatment effects on state anxiety, we conducted an analysis of variance (ANOVA) with repeated measures on phase (anxiety_base_, anxiety_stress_) and time (pre-treatment/waiting, post-treatment/waiting), using group (CBT, WLC) as a between-subjects factor and anxiety after the social stress task as dependent variable.^5^ For treatment effects on cognitions, we conducted a multivariate ANOVA (MANOVA) with repeated measures on time (pre-treatment/waiting, post-treatment/waiting), using group (CBT, WLC) as a between-subjects factor and SISST-PS scores (negative, positive) as dependent variables. For treatment effects on behavior, we conducted a MANOVA with repeated measures on time (pre-treatment/waiting, post-treatment/waiting), using group (CBT, WLC) as a between-subjects factor and PQ-C scores (microbehaviors, nervousness, global impression) as dependent variables. For treatment effects on physiology, we conducted an ANOVA with repeated measures on time (pre-treatment/waiting, post-treatment/waiting), using group (CBT/WLC) as a between-subjects factor and heart rate scores for narrating the story and performing the calculation as dependent variables.

#### Diagnostic Data

Age and gender were included as covariates as these have previously been identified as potential influences on SAD symptoms [55] and treatment success [21, 22].

The main analyses of the SPAI-C, SASC-R_child report_, and SASC-R_parent report_, were conducted with the open-source statistical software R, using the mixed-models packages lme4 [65] and lmerTest [66]. These models were fitted with one between-subjects factor, group (levels: CBT, WLC), one within-subject factor, time (levels: admission, pre-treatment/waiting, post-treatment/waiting), and the interaction between group and time as fixed effects. Furthermore, intercepts for every participant were modeled as random effects. All degrees of freedom were calculated with Satterthwaite approximation. There is an ongoing debate about effect sizes in mixed models. However, no consensus has yet been achieved and thus no effect size can be reported [67].

Significant main effects and interactions for all ANOVAs and MANOVAs were further analyzed with post hoc *t* tests for independent groups for the group comparisons and with *t* tests for dependent groups for the time comparisons if relevant for the hypotheses. Trends for significant effects (*p* < 0.10), if in line with the direction of the hypotheses, are reported but interpreted with caution. Cohen’s *d* effect sizes are reported for the post hoc tests.

The analysis of the diagnostic interviews was conducted in R using the nparLD package [68] for nonparametric longitudinal data with group (levels: CBT, WLC) as a between-subjects factor and time (levels: pre-treatment/waiting, post-treatment/waiting) as within-subject factor as well as their interaction. A nonparametric analysis was chosen as severity scores are ordinally scaled. Significant interactions were further explored with nonparametric Mann–Whitney *U* tests. Additionally, the diagnostic status after treatment and waiting, respectively, in each group (CBT, WLC) was analyzed with a χ^2^ test.

## Results

### Changes in Laboratory Data Before and After Intervention

For state anxiety, the MANOVA with repeated measures revealed a significant main effect of phase during the TSST-C, a significant main effect of time between TSST-C 1 and 2, but no main effect of group. Furthermore, the Time × Phase interaction reached significance, indicating a change in anxiety reactivity from TSST-C 1 to TSST-C 2. The Time × Group interaction showed a trend toward significance, and the Time × Phase × Group interaction remained nonsignificant (see Table 2). As all effects relevant to the hypotheses remained nonsignificant, no post hoc tests were conducted.

**Table 2:** Results changes in laboratory data before and after intervention

Variable	Wilk’s λ	*df*	*F*	*p*	η_p_^2^
State anxiety					
Phase	0.299	1.55	129.19	< .001	0.701
Time	0.815	1.55	12.49	0.001	0.185
Group		1.55	2.39	0.128	0.042
Time × Phase	0.922	1.55	4.64	0.036	0.078
Time × Group	0.948	1.55	3.04	0.087	0.052
Time × Phase × Group	0.998	1.55	0.1	0.749	0.002
Cognitions					
Time	0.858	2.48	3.98	0.025	0.142
Group	0.974	2.48	0.65	0.529	0.026
Time × Group	0.874	2.48	3.46	0.04	0.126
Social performance					
Time	0.939	3.43	0.93	0.435	0.061
Group	0.915	3.43	1.33	0.277	0.085
Time × Group	0.981	3.43	0.28	0.837	0.019
Heart rate					
Time	0.875	2.38	2.72	0.078	0.125
Group	0.92	2.38	1.66	0.204	0.08
Time × Group	0.95	2.38	0.99	0.379	0.05

**Fig. 3:**
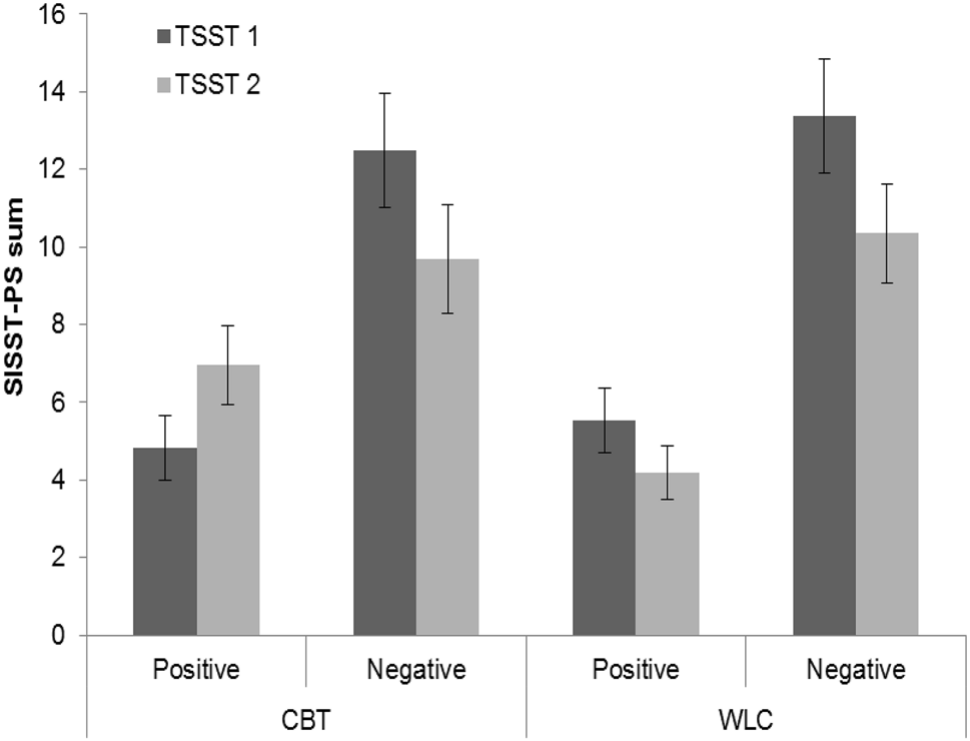
Positive and negative cognitions before (TSST-C 1) and after (TSST-C 2) treatment or waiting. *SISST-PS* Social Interaction Self-Statement Test-Public Speaking, *CBT* cognitive behavioral therapy group, *WLC* waitlist control group

For cognitions, the MANOVA with repeated measures showed a significant main effect of time but not group. A significant Time × Group interaction was found (see Table 2). This interaction was significant for positive cognitions, *F*_(1,49)_ = 6.19, *p* = 0.016, $\eta_{\text{p}}^{2}$ = 0.112, but not for negative cognitions, *F*_(1,49)_ = 0.22, *p* = 0.640, $\eta_{\text{p}}^{2}$ = 0.005 (see Table 3 for means and standard deviations). On the basis of this finding, we conduced post hoc *t* tests for dependent groups for positive cognitions, which showed a trend toward a significant increase in positive cognitions in the CBT group, *t*_(25)_ = 1.91, *p* = 0.068, *d* = 0.37, and a trend toward a significant decrease in positive cognitions in the WLC group, *t*_(25)_ = − 2.05, *p* = 0.051, *d* = − 0.40 (Fig. 3).

**Table 3:** Laboratory data pre/post treatment vs. waiting

Variable	pre			post		
	CBT	WLC	Statistics	CBT	WLC	Statistics
State anxiety	6.7 (2.93)	6.6 (2.76)	*p* = .920	6.7 (2.82)	5.5 (3.68)	*p* = .189
Cognitions						
Positive	4.8 (4.34)	6.8 (4.96)	*p* = .089	7.1 (5.92)	4.7 (4.49)	*p* = .096
Negative	12.6 (7.56)	12.9 (7.67)	*p* = .897	9.8 (7.00)	10.1 (6.43)	*p* = .859
Social performance						
Microbehaviors						
Nervousness	3.5 (1.46)	3.0 (1.66)	*p* = .260	3.8 (1.85)	3.0 (1.74)	*p* = .118
Global impression	4.2 (1.88)	4.2 (2.24)	*p* = .984	4.0 (1.84)	3.4 (1.74)	*p* = .171
	2.4 (1.96)	2.1 (1.55)	*p* = .508	2.8 (1.99)	2.1 (1.75)	*p* = .185
Heart rate						
Story	96.7 (15.52)	92.9 (11.13)	*p* = .307	99.5 (16.21)	98.0 (13.95)	*p* = .752
Calculation	92.8 (13.29)	88.0 (9.27)	*p* = .127	94.2 (14.80)	90.6 (10.17)	*p* = .366

For social performance, the MANOVA with repeated measures revealed no significant main or interaction effects, all *F*s < 1.33, all *p*s > 0.276. For heart rate, the MANOVA with repeated measures revealed no significant main or interaction effect, all *F*s < 2.73, all *p*s > 0.077 (see Tables 2 and 3).

### Changes in Diagnostic Data: Intervention Effects as Measured by Social Anxiety Symptoms

#### Child Report

**Fig. 4:**
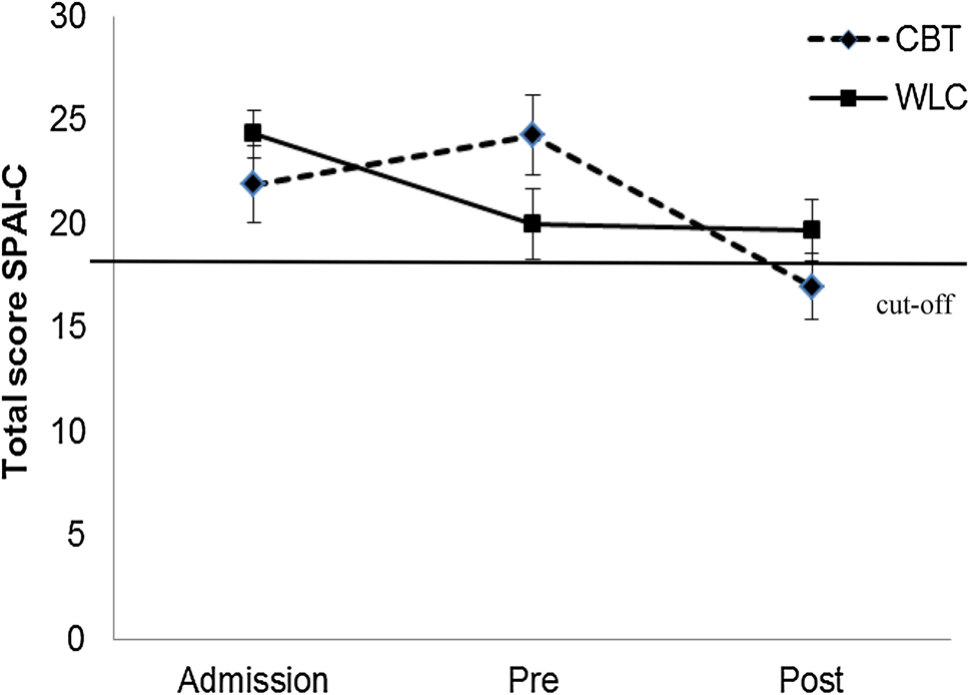
Social Phobia and Anxiety Inventory for Children (SPAI-C) course from admission to pretreatment/waiting (pre) to posttreatment/waiting (post) in the cognitive behavior therapy (CBT) treatment group and the waitlist control (WLC) group, including clinical cut-off at 18

The mixed-models analysis of social anxiety symptoms reported by the child (SPAI-C) showed a significant main effect of time, *F*_(2,116.6)_ = 10.86, *p* < 0.001, but no main effect of group, *F*_(1,65.2)_ = 0.02, *p* = 0.899.^6^ Additionally, a significant interaction effect of Time × Group was found, *F*_(2,116.6)_ = 5.87, *p* = 0.004. Post hoc paired *t* tests (two tailed) revealed a significant difference in the CBT group between admission and pre-treatment/waiting, *t*_(27)_ = -2.06, *p* = 0.049, *d* = 0.39, as well as between pre-treatment/waiting and post-treatment/waiting, *t*_(27)_ = 4.68, *p* < 0.001, *d* = 0.89 (see Fig. 4). In the WLC group, a difference was found between admission and pre-treatment/waiting, *t*_(26)_ = 2.65, *p* = 0.014, *d* = 0.51, but not between pre-treatment/waiting and post-treatment/waiting, *t*_(25)_ = 1.37, *p* = 0.182. Thus, in the crucial phase of treatment/waiting (pre to post), social anxiety scores decreased only in the treatment group.

A similar approach was used for social anxiety symptoms reported in the SASC-R_child report_, which showed a significant main effect of time, *F*_(2,115.6)_ = 10.31, *p* < 0.001, but no main effect of group, *F*_(1,66)_ = 0.39, *p* = 0.534. Furthermore, the interaction effect Time × Group did not reach significance, *F*_(2,115.6)_ = 1.16, *p* = 0.316. The SASC-R_child report_ score at admission and pre-treatment/waiting did not differ, *t*_(53)_ = 0.09, *p* = 0.379, *d* = 0.12, but it decreased significantly between pre-treatment/waiting and post-treatment/waiting, *t*_(53)_ = 3.67, *p* < 0.001, *d* = 0.50. As the course of social anxiety did not differ between groups, no post hoc tests between groups were performed.

#### Parent Report

A similar approach based on mixed models was used for the analysis of parent reports of child social anxiety symptoms (SASC-R_parent report_). This revealed a main effect of time, *F*_(2,114.4)_ = 7.23, *p* = 0.001, but no main effect of group, *F*_(1,65.2)_ = 0.27, *p* = 0.608. Further, the interaction effect Time × Group did not reach significance, *F*_(2,114.4)_ = 1.01, *p* = 0.366. The SASC-R_parent report_ score in both groups did not change between admission and pre-treatment/waiting, *t*_(53)_ = − 1.11, *p* = 0.272, but decreased significantly between pre-treatment/waiting and post-treatment/waiting, *t*_(50)_ = 3.95, *p* < 0.001, *d* = 0.55. As the course of social anxiety did not differ between groups based on parent report, no post hoc tests were performed.

Further analyses for the net therapy effect at post-treatment and follow-up showed a significant decrease in child and parent reports of social anxiety symptoms over time, *p*s < 0.001. Thus, treatment effects continued even after treatment had stopped. Detailed analyses are reported in the online supplements.

#### Changes in Diagnostic Data: Intervention Effects as Measured by a Structured Interview

**Fig. 5:**
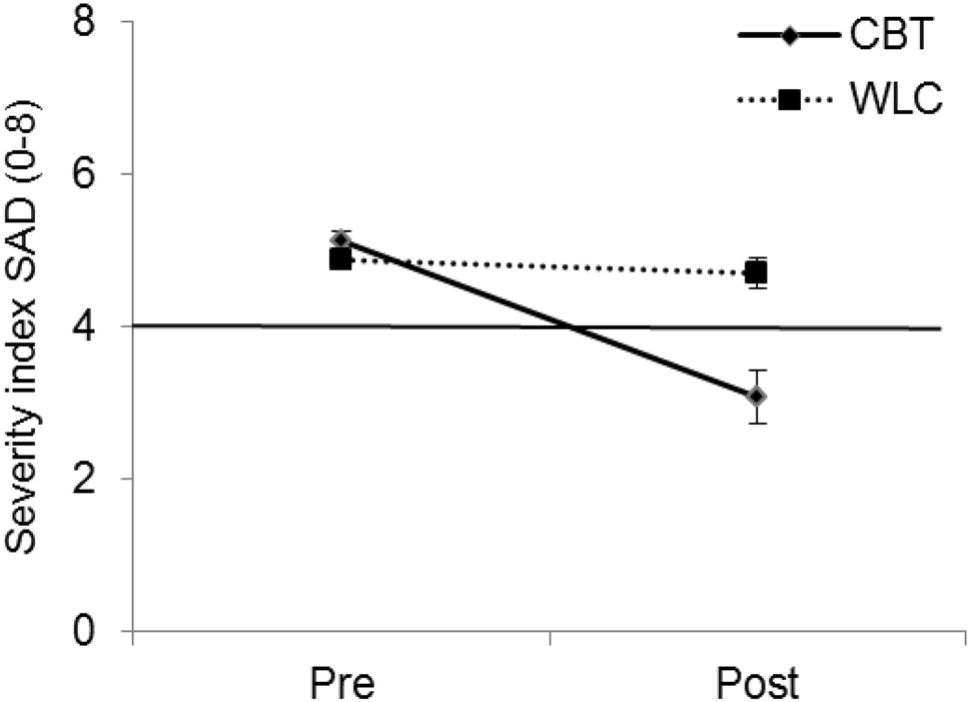
Severity scores pretreatment/waiting and posttreatment/waiting in the treatment group (CBT) and the waitlist control (WLC) group, including clinical cut-off at 4

We performed an analysis with the severity of the SAD diagnosis as assessed by a structured interview blind to treatment condition as the dependent variable, again using the factors group (CBT, WLC) and time (pre-treatment/waiting, post-treatment/waiting). It showed a significant main effect of time, *F*_(1)_ = 28.68, *p* < 0.001, and group, *F*_(1)_ = 7.24, *p* = 0.007. Additionally, the interaction effect Time × Group was significant, *F*_(1)_ = 16.23, *p* < 0.001. Children in the CBT and WLC groups did not differ in severity of the SAD diagnosis before treatment/waitlist, *Z* = 1.52, *p* = 0.127. The severity decreased significantly in the CBT group while remaining stable in the WLC group, *Z* = 3.95, *p* < 0.001 (see Fig. 5).^7^

This result was confirmed with a comparison of diagnostic status in the two groups, showing a significant difference between the CBT and WLC groups after treatment and waiting, respectively, χ^2^(1) = 6.09, *p* = 0.014. While no child in the WLC group was diagnosis free after treatment (0%), several children in the CBT group were no longer diagnosed with SAD (26%).

## Discussion

This study aimed to extend findings from a previous study [36] on the efficacy of a SAD-specific group CBT by assessing change not only with parent and child reports but also with clinical interviews and a social stress test. It should be cautioned that affective, behavioral, and physiological responses during high social stress did not change as a result of treatment, thus indicating the situation was still highly stressful. However, a significant interaction between time and group appeared for positive cognitions, which resulted in a trend-significant increase of positive cognitions in children in the CBT group from pre- to post-treatment. This finding is in line with previous findings of a positive CBT effect on cognitions after a strong focus on exposure [23] and supports theories of the importance of cognitions in both the stability [50, 69, 70] and the treatment [71, 72] of the disorder. As state social anxiety values indicate, the experience of social stress remained high. Nevertheless—keeping in mind the trend significance—we can assume that children were able to think more positively about the situation (e.g., “It might be embarrassing, but I can cope with the situation”). One could assume that cognitive changes occur before other factors innate to SAD follow, such as the perception of behavior or a physiological response [72]. However, this mediational assumption needs further research. For the physiological response, it should be kept in mind that most research is inconclusive about an objective hyperarousal before treatment and it further tends to support the idea of a biased perception of an increased heart rate [30, 73]. Our findings show that even in the CBT group, the heart rate during social stress still increased compared to a baseline measure. While this might be a normal stress response [45], further studies are needed to clarify the stability of this result. Similar to the physiological results, those on behavioral measures such as social performance indicate that it is more the perception of social performance than the social performance itself that is decreased in children with SAD [33, 61]. Considering these findings, we expected an improvement in perception of social performance. Even though social skills and positive self-feedback were trained in role-playing exercises in a group with peers during CBT treatment, this perception did not change in the CBT group. Our findings do not allow direct assumptions about the cause of this lack of change. It could be that treatment was too short, as only repeated exposure over a long time and—possibly—increased positive social feedback lead to a change in this bias [7].

For diagnostic measures, a significant decrease appeared in child-reported social anxiety as assessed with the SPAI-C, but not when assessed using the SASC-R. Although both questionnaires assess typical dimensions of SAD, they have different foci: The SPAI-C assesses behavioral, physiological, and cognitive features across different types of social situations, while the SASC-R is more narrowly focused on fear of negative evaluation and social distress experience. It may be that—similar to the laboratory response—children rather perceived change in their cognitions than in other associated symptoms that are measured by the SASC-R. Quite a large number of children still met a diagnosis of SAD after treatment, which is in line with previous studies [7]. However, responder analyses based on full remission should be considered carefully because of, for example, their lack of power [74].

Extending the findings of an earlier study [36], the decrease in the severity index of the SAD diagnosis coded by blind interviewers supports the efficacy of the current group treatment. In the months following treatment, anxiety symptoms further decreased continuously on all three questionnaires (see online Supplement 1). In contrast to the earlier study [36], no decrease in dimensional social anxiety symptoms was found for the parent report, but a decrease was found for the child report. One possible explanation could be the slightly greater average age of the children in the current study. Insight into the relationship between anxiety (i.e., emotion) and avoidance (i.e., behavior or coping) is still limited in younger children [75]. Therefore, the significant reduction in child-reported SAD symptoms could be a result of older children’s greater cognitive insight. They might acknowledge both avoidance and anxiety (“I am afraid of others, so I do not talk to them”), and treatment helps them engage in less avoidance (“Even though I am afraid of others, I do talk to them”). While self- and parent-reported anxiety symptoms are both important criteria for treatment success, their reliability can be questioned, as several studies have found inconsistencies between parent- and child-reported anxiety [76]. Still, disagreements might be the result of different perspectives (e.g. [77].); in the current case, children may have already perceived a change in their SAD symptoms while parents had not yet rated these as substantial (possibly because they were not present during treatment). We have to acknowledge that questionnaires allow for only a limited assessment of SAD’s multiple facets. Thus, combined with our laboratory findings, results of the diagnostic measures indicate it might be possible that children still experience anxiety after treatment but have learned to cope better with their anxiety.

Even though CBT—in both group and individual set-up [14]—is the gold standard treatment for anxiety disorders, outcomes for SAD have repeatedly been shown to be inferior to outcomes for other anxiety disorders (e.g. [7].). One possible way to improve outcomes might be to extend treatment to more exposure sessions (see [78]). This recommendation is based on issues concerning the treatment of both temperamental, that is, behavioral inhibition, and interactional difficulties [4]: First, behaviorally inhibited children and youths—even before developing SAD—behave in a socially avoidant manner from an early age and as such might lack experience approaching social situations. Because of this avoidance, they receive limited positive social feedback, and anxiety in unstructured social interactions increases. Second, a general first tendency not to actively approach social situations might lead to a social skills deficit. A vicious circle can develop in which deficits in social skills increase the chance of negative social outcomes. Subsequently, expected negative outcomes of future social situations lead to thoughts of social inadequacy and the avoidance of social situations. Thereby, the development of social skills is further impeded, as few opportunities arise to practice coping with social situations [79]. Even though a social skills deficit is not apparent in all patients with SAD [60], the subjective perception of oneself as being (socially) incompetent might lead to further increases in anxiety and subsequent difficulties in social performance. Thus, given this lengthy developmental process and complex interactional demands, treatment over 12 sessions targeting all deficient elements (social skills deficits, negative cognitions, avoidance of social situations, etc.) can only be seen as an initiation of change. An efficient solution to enhance treatment effects could be a booster session model that allows flexible extension of treatment for those who need more than standard treatment (e.g. [80, 81].). Furthermore, a break after the first 12 sessions would allow treatment effects to stabilize in everyday life. The booster sessions would then provide the possibility to refresh learned skills to avoid relapse to old avoidant behavior.

While our study was carefully planned, several limitations apply. A comparison to individual treatment, not examined in this study, should be examined in future research. Previous studies comparing individual to group CBT did not show a clear preference for either [13, 14]. Still, our aim was not to demonstrate the superiority of group CBT over individual CBT but rather to provide empirical evidence for an efficient group treatment program. Additionally, the TSST-C is a highly potent stressor and, therefore, possibly not the best choice to examine treatment success. Previous studies with adult participants showed that even healthy people do not easily adapt or habituate to a second exposure to the TSST (for an overview see [45]). As mentioned before, it is even more remarkable that our results can be cautiously interpreted into the direction that children with SAD were able to change their cognitive coping with this highly stressful situation. To understand moderators and mediators of change, a follow-up TSST or other social stress task could provide insight on the mechanisms: Possibly, cognitions change after treatment while changes in behavioral, physiological, and affective factors follow several months later.

In treatment research, more randomized controlled trials including experimental designs are necessary to shed further light on the current findings, possibly varying setting (single vs. group therapy) and parental inclusion. Further, measures of success across studies differ widely even when targeting only disorder-specific psychopathology, with both general [22] and specific [21, 82] anxiety questionnaires having been used. The gold standard of a blind diagnostic interview before and after treatment should be applied to both parents and children to include both perspectives in the quality assessment of treatment.

## Summary

The study aimed to assess CBT treatment success of child SAD not only by social anxiety reports but also by cognitive, behavioral, and physiological components of social stress. Children with SAD participated in a standardized social stress test before and after treatment or a waitlist control period. The CBT group showed a trend toward a significant increase in positive cognitions under social stress after treatment, while these cognitions decreased in the WLC group. No significant results appeared for behavior and physiology. Children in the CBT group, but not parents, further reported less social anxiety in one questionnaire from pre- to post-treatment. A structured interview confirmed a decrease in severity of SAD in the CBT group. While the gold standard of a blind interview showed efficacy of treatment, not all trait and state measures demonstrated similar success patterns. Therefore, this randomized controlled trial of an exposure-based treatment approach in a group setting showed this treatment as partly effective intervention for childhood SAD. A strong focus on exposure produced a trend toward significant change in cognitions during socially stressful situations. However, modifications of both the treatment group setting and the assessment of outcomes, including the use of multiple measures of social anxiety and experimental paradigms, warrant further research. Treatment of SAD needs etiologically based interventions, and possible effective modules in addition to exposure remain to be empirically verified.

## Electronic supplementary material

Below is the link to the electronic supplementary material.

Electronic supplementary material 1 (DOCX 31 kb)

Electronic supplementary material 2 (DOCX 77 kb)

## Footnotes

1 This comprehensive project focused on affective, cognitive, and physiological processes of social anxiety in children. For each domain, multiple variables were assessed. Some of these were a priori defined as later outcome measures (as the focus of the project was to elaborate on these basic processes and subsequently assess treatment efficacy, not all assessed variables from those basic processes were designed as outcome measures and some of the assessed variables were conceptualized as predictors of treatment outcome instead of a primary target of change). However, these basic processes may also help explain the process of change. Therefore, we outline in each manuscript related to this project that concerns change processes if the respective variables were a priori defined as outcome variables or post hoc explored for their relevance to change. Furthermore, not all outcome measures could be reported in one outcome manuscript due to constraints in length. The outcome papers were divided by primary topic into the current article (state anxiety, negative cognitions, physiological arousal) and other articles (perception of and worry about physiological symptoms, perception of academic performance, negative post-event processing, parental cognitions). Results are reported elsewhere (post-event processing, [83]; cortisol, [84] or are being prepared for submission. All articles include cross-references to other outcome measures.

2 All analyses first considered site differences, which were non-existent.

3 A preliminary manipulation check was conducted to compare children with SAD to healthy control children. Compared to healthy control children, children in the SAD group reported more negative cognitions, *F*_(1,109)_ = 34.41, *p* < .001, η_p_^2^ = .240, while no differences emerged for positive cognitions, *F*_(1,109)_ = 0.06, *p* = .812, η_p_^2^ = .001. Concerning behavioral measures, children in the SAD group had lower scores in microbehaviors, *F*_(1,105)_ = 11.08, *p* = .001, η_p_^2^ = .095, and global impression, *F*_(1,105)_ = 11.82, *p* = .001, η_p_^2^ = .101, but higher scores in nervousness, *F*_(1,105)_ = 8.86, *p* = .004, η_p_^2^ = .078. Children with SAD and healthy control children did not differ concerning heart rate measures, *F* < 0.58, *p* > .565.

4 Statistical outliers 2.5 *SD* above or below the mean were excluded. Outliers were calculated separately for groups and time.

5 Due to single missing values, analyses were not conducted with all participants (included *n*—state anxiety: CBT = 30, WLC = 27; cognitive data: CBT = 26, WLC = 25; behavior data: CBT = 27, WLC = 20; physiology: CBT = 26, WLC = 19).

6 Age, sex, and center effects were modeled as fixed factors. As none of them added significant value to any of the analyzed models, all *p*s > .05, they are not included in the final presentation of results.

7 No differences in the SAD severity index were observed between children with and without comorbid disorders pre and post therapy.
