# Supplementary material for: Experience Versus Report: Where Are Changes Seen After Exposure-Based Cognitive-Behavioral Therapy? A Randomized Controlled Group Treatment of Childhood Social Anxiety Disorder
Source: Child Psychiatry Hum Dev. 2020 Jan 20;51(3):427–41. doi: 10.1007/s10578-019-00954-w (PMC7235054; doi:10.1007/s10578-019-00954-w)
Supplement: Supplementary file 1 — Electronic supplementary material 1 (DOCX 31 kb) [file 10578_2019_954_MOESM1_ESM.docx]

**Net Therapy effects**

**Statistical analyses**

To estimate the net therapy effect at post-treatment and follow-up, additional analyses using a mixed models approach with factors group (levels: EG / WLC) and time (levels: PRE / POST / Follow-Up 1 [FU1] / Follow-Up [FU1]) were performed. The time course of the WLC was shifted by one, in order to achieve modelling synchronicity between the CBT and the WLC groups. Therefore, non-significant time×group interactions indicate the same therapeutic effect in both groups across equal follow-up time courses. SPAI-C, SASC-Child and SASC-Parents net therapy and follow-up effects were modelled with one between subjects factor group (levels: CBT / WLC), one within subject factor time (levels: pretreatment/waiting, posttreatment/waiting, follow-up 1, follow-up 2), and the interaction between group and time as fixed effects. Intercepts for every subject were modelled as random effects.

**Results**

### Diagnostic data: Overall stability of treatment effects.

*Child report*. The mixed models analysis of social anxiety symptoms reported by the child (SPAI-C) based on the factors group (levels: CBT / WLC) and time (levels: pretreatment/waiting [PRE], posttreatment/waiting [POST], follow-up 1 [FU1], follow-up 2 [FU2) revealed a significant main effect of time, *F*_(3,135.9)_ = 25.94, *p* < . 001, but neither a significant main effect of group, *F*_(1,58.9)_ = 2.29, *p* = .135, nor an interaction effect time × group, *F*_(3,135.9)_ = 1.25, *p* = .293. Thus, as expected, the groups did not differ over time but social anxiety symptoms steadily decreased. Post-hoc paired *t* tests (two-tailed) showed a significant decrease in symptoms from PRE to POST, *t*_(48)_ = 5.11, *p* < .001, *d* = 0.73, and from POST to FU1, *t*_(43)_ = 2.32, *p* = .025, *d* = 0.35, while no difference appeared between FU1 and FU2, *t*_(36)_ = 1.24, *p* = .224 (see Figure S1).

cut-off

Figure S1. SPAI-C course from pre-treatment (PRE) to post-treatment (POST) to follow-up 1 (FU1; 3 months) and follow-up 2 (FU2; 6 months) including both groups’ treatment phase (with the horizontal line representing the clinical cut-off of 18).

A similar approach for social anxiety symptoms as reported in the SASC-R-D_(child report)_ showed a significant main effect of time, *F*_(3,138.1)_ = 13.91, *p* < .001, but neither a main effect of group, *F*_(1,59.8)_ = 1.53, *p* = .221, nor an interaction effect time × group: *F*_(3,138.1)_ = 2.06, *p* = .108. Thus, again as expected, the groups did not differ over time but social anxiety symptoms steadily decreased. Post-hoc paired *t* tests (two-tailed) showed a significant decrease in symptoms from PRE to POST, *t*_(48)_ = 2.47, *p* = .017, *d* = 0.35, and from POST to FU1, *t*_(44)_ = 3.33, *p* = .002, *d* = 0.50, while no difference appeared between FU1 and FU2, *t*_(36)_ = 0.97, *p* = .339 (see Figure S2).

cut-off boys

cut-off girls

Figure S2. SASC-R course as reported by child and parent from pre-treatment (Pre) to post-treatment (Post) to follow-up 1 (FU1; 3 months) and follow-up 2 (FU2; 6 months) including both groups’ treatment phase and clinical cut-off.

*Parent report*. Again a similar approach based on mixed models was used for the analysis of parental report of child social anxiety symptoms (SASC-R-D_(parent report)_). This analysis revealed a main effect of time, *F*_(3,144.3)_ = 18.02, *p* < .001, but neither a main effect of group: *F*_(1,63.7)_ = 1.18, *p* = .282, nor an interaction effect time × group, *F*_(3,144.3)_ = 1.29, *p* = .282. Thus, in parallel to child report, the groups did not differ over time but social anxiety symptoms steadily decreased. Post-hoc paired *t* tests (two-tailed) showed a significant decrease in symptoms from PRE to POST, *t*_(47)_ = 3.42, *p* = .001, *d* = 0.49, and from POST to FU1, *t*_(47)_ = 2.40, *p* = .020, *d* = 0.35, while no difference appeared between FU1 and FU2, *t*_(39)_ = 0.97, *p* = .340 (see figure S2).
