# Supplementary material for: Experience Versus Report: Where Are Changes Seen After Exposure-Based Cognitive-Behavioral Therapy? A Randomized Controlled Group Treatment of Childhood Social Anxiety Disorder
Source: Child Psychiatry Hum Dev. 2020 Jan 20;51(3):427–41. doi: 10.1007/s10578-019-00954-w (PMC7235054; doi:10.1007/s10578-019-00954-w)
Supplement: Supplementary file 2 — Electronic supplementary material 2 (DOCX 77 kb) [file 10578_2019_954_MOESM2_ESM.docx]

**Emotion regulation and self-attention**

## Psychometric Measures

*Emotion Regulation Questionnaire state (ERQ-S,* [1]. The ERQ-S assesses ER strategies in a current situation based on Gross’ (1998) model of emotion regulation. It was modified from an instrument used in research with adults to assess reappraisal and suppression using 6 items. While psychometric properties were sufficient in an adult sample [1], in our sample of children sufficient internal consistency was only found for reappraisal in both testing sessions (both α = .72). Internal consistency for suppression was not acceptable (α_1_ = -.53, α_2_ = -.14). Further principal component analyses suggested the problem could be attributed to one inconsistent item (“I tried to control my emotions”). Thus, after including only the remaining two items, internal consistency improved significantly (α_1_ = .83, α_2_ = .55).

*Self-Attention Questionnaire.* We developed a short questionnaire measuring self-attention, which was administered three times during the session. It was based on previous measures on self- and other-focused attention [2, 3]. Children reported, across seven items, how much they focused on their body (e.g. “I focused on how my voice sounded”), their thoughts (e.g., “I focused on what was going on inside me”) and their general impression (e.g., “I focused on the impression I made”) on a 5-point Likert scale (0 = “not at all” to 4 = “all the time”). Internal consistency ranged from good to excellent at all measurements (α_n_ = .83 - . 91).

## Statistical Analyses

Effects on self-attention were analyzed using an ANOVA with independent variable *group* (CBT, WLC) with repeated measures on *session* (PRE, POST) and *phase* (baseline, stress, recovery). Similarly, effects on emotion regulation, a multivariate ANOVA with independent variable *group* (CBT, WLC) with repeated measures on *session* (PRE, POST) and dependent variables *strategies* (reappraisal, suppression) was calculated. Significant main effects and interactions were further analyzed with t-tests for independent groups for the *group* comparisons and with t-test for dependent groups for the *session* comparisons^[[1]](#footnote-1)^.

# Results

## Self-attention.

The analysis of self-attention after treatment showed significant main effects of *session*, *F*_(1,41)_ = 4.64, *p* = .037, η_p_^2^ = .102, and *phase*, *F*_(2,40)_ = 34.67, *p* < .001, η_p_^2^ = .634. All other effects (*group*, all interaction effects) were non-significant, *F*s < 1.84, *p*s > .171. Similar to the first session, dependent t-tests for phase in the second session showed a significant increase overall between baseline and stress, *t*_(49)_ = -6.62, *p* < .001, *d* = -0.94, and a significant decrease between stress and recovery, *t*_(48)_ = 6.27, *p* < .001, *d* = 0.90. Thus, while self-attention reports decreased on average regardless of treatment, children still reported significant changes in self-attention during confrontation with a social stressor (see Figure S2.1).

CBT

Figure S2.1. No effects of CBT on self-attention reported by children during the TSST-C before (_1) and after (_2) treatment.

## Emotion regulation.

The analysis of emotion regulation after treatment showed significant main effects of *strategy*, *F*_(1,54)_ = 61.60, *p* < .001, η_p_^2^ = .533, and *session*, *F*_(1,54)_ = 5.49, *p* = .023, η_p_^2^ = .092, while group remained non-significant, *F*_(1,54)_ = 0.47, *p* =.498, η_p_^2^ = .009. Further, significant interaction effects were found for *strategy x group*, *F*_(1,54)_ = 4.43, *p* = .040, η_p_^2^ = .076, *group* *x session,* *F*_(1,54)_ = 10.09, *p* = .002, η_p_^2^ = .157, *strategy x session, F*_(1,54)_ = 4.09, *p* = .048, η_p_^2^ = .070, and the 3-way interaction *group x strategy x session,* *F*_(1,54)_ = 4.94, *p* = .031, η_p_^2^ = .084.

Follow-up univariate ANOVAs disentangling both strategies showed significant effects for reappraisal for *session*, *F*_(1,55)_ = 8.30, *p* = .006, η_p_^2^ = .131, *group*, *F*_(1,55)_ = 12.94, *p* = .001, η_p_^2^ = .190, and the interaction *group x session*, *F*_(1,55)_ = 12.94, *p* = .001, η_p_^2^ = .190. All effects for suppression were non-significant, *F*s < 0.82, *p*s > .371. Thus, reappraisal increased only in the CBT (see Figure S2.2).


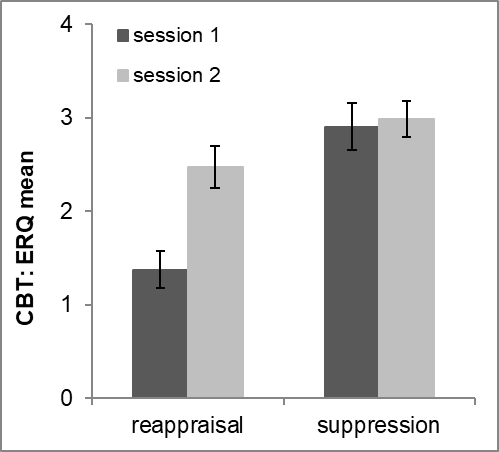

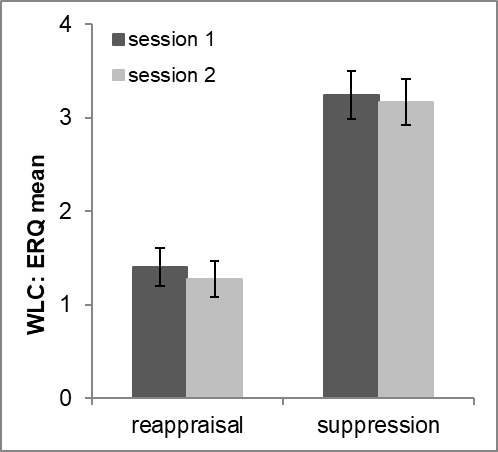


**B**

**A**

Figure S2.2. Change of reappraisal but not suppressive strategies in CBT group (A). Lack of change in both ER strategies in waitlist control group (B).

# References

1. Egloff B, Schmukle SC, Burns LR, Schwerdtfeger A. Spontaneous emotion regulation during evaluated speaking tasks: associations with negative affect, anxiety expression, memory, and physiological responding. Emotion. 2006;6:356–66.

2. Woody S, Rodriguez B. Self-focused attention and social anxiety in social phobics and normal controls. Cognit. Ther. Res. 2000;24:473–88.

3. Kley H, Tuschen-Caffier B, Heinrichs N. Safety behaviors, self-focused attention and negative thinking in children with social anxiety disorder, socially anxious and non-anxious children. J. Behav. Ther. Exp. Psychiatry. 2012;43:548–55.

4. Mathews BL, Koehn AJ, Abtahi MM, Kerns KA. Emotional competence and anxiety in childhood and adolescence: A meta-analytic review. Clin. Child Fam. Psychol. Rev. 2016;19:162–84.

5. Compas BE, Jaser SS, Bettis AH, Watson KH, Gruhn M a, Dunbar JP et al. Coping, emotion regulation, and psychopathology in childhood and adolescence: A meta-analysis and narrative review. Psychol. Bull. 2017;143:939–91.

1. Several authors (e.g., Compas et al., 2017; Mathews et al., 2016) emphasize the importance of age in consideration of ER as the process may change during child development. However, inclusion of age as a covariate did not change the current results and was thus omitted from further report. [↑](#footnote-ref-1)
